# Sensing Properties of Fused Silica Single-Mode Optical Fibers Based on PPP-BOTDA in High-Temperature Fields

**DOI:** 10.3390/s19225021

**Published:** 2019-11-18

**Authors:** Jiahui Shen, Ting Li, Hong Zhu, Caiqian Yang, Kai Zhang

**Affiliations:** 1 Key Laboratory of Concrete and Prestressed Concrete Structures of the Ministry of Education, Southeast University, Nanjing 210096, China; shenjiahui2018@126.com (J.S.); litingyxsh@163.com (T.L.); ycqjxx@hotmail.com (C.Y.); 2 Nanjing Institute of Underground Space Advanced Technology, Nanjing 210032, China; 3 HUALU Engineering and Technology Co., Ltd., Xian 710065, China; zk2657@hlet.com

**Keywords:** self-sensing property, temperature coefficient, strain coefficient, high-temperature experiments, FRP

## Abstract

The strain of fiber-reinforced polymer (FRP) bars at high temperatures is currently difficult to measure. To overcome this difficulty, a method of smart FRP bars embedded with optical fibers was proposed and studied, in which an ordinary single-mode optical fiber was applied as a distributed sensor. In this paper, both the distributed temperature and strain-sensing characteristics of optical fiber were studied based on pulse pre-pump Brillouin optical time-domain analysis (PPP-BOTDA) under high temperature. The temperature and strain coefficients were investigated under a thermomechanical coupling environment with consideration of large strain levels. The experimental results show that the temperature and strain coefficients decreased as the temperature increased, because the properties of silica and coating materials changed with temperature. Then, the formulas for determining the temperature and strain coefficients at high temperatures were introduced and discussed. The excellent sensing performance of the optical fiber indicated that smart FRP bars have the potential for use at high temperatures.

## 1. Introduction

Fiber-reinforced polymer (FRP) bars, as advanced materials, are expected to be a promising substitute for steel bars. Due to having several superior characteristics, FRP bars have attracted increasing attention and have been generally used for new concrete structures or for structural reinforcement. To evaluate the structural performance, the characteristics of the bars in all kinds of conditions should be well known. When experiments are conducted at room temperature, the strain of the bars can be easily monitored by conventional strain gauges. However, when in fire or a high-temperature environment, these strain gauges rapidly fail because they are not immune to high temperatures. As a result, strain gauges cannot meet the reliable measurement requirement. Therefore, a more effective method of monitoring is urgently needed to solve this problem.

In recent years, structural health monitoring (SHM) has played a significant role in detecting damage characteristics in structures, such as cracks, fatigue, and slip [1]. Among the methods used, fiber optic sensors have gained increasing attention. Fiber optic sensors have been widely applied in many fields, such as airplanes/spacecraft [2], piles [3,4], civil infrastructure [5,6], submarines and deep-sea floor [7,8], health care [9], etc. After decades of development, two types of optical fiber sensing technology have been utilized or even improved upon in lab experiments and actual civil engineering, as follows: Fiber Bragg grating (FBG) and Brillouin scattering based optical sensors. FBG sensing is a typical “point” sensing technique that allows distribution through an array of FBG sensors [10], whereas Brillouin optical sensing is a truly distributed technique. Generally, Brillouin optical sensing includes Brillouin optical time-domain analysis (BOTDA) and Brillouin optical time-domain reflectometry (BOTDR), and this method is convenient and economical. In particular, improved BOTDA, i.e., pre-pump pulse BOTDA (PPP-BOTDA), has higher static monitoring accuracy for temperature and strain, as well as sensing frequency [11]. Therefore, it is advisable to use optical fiber in measuring temperature and strain of FRP at high temperatures, based on PPP-BOTDA. However, it is necessary to embed optical fibers into FRP bars to fabricate smart bars so that FRP can provide the sensors with reliable and durable protection, while the sensors also provide the FRP with a self-monitoring function [12,13,14]. A previous study showed that smart FRP bars have been applied to many areas of concrete structures [15].

However, regardless of the kind of sensor, the monitoring was mostly carried out at or above room temperature but below the 100 °C reported in previous studies [15,16], which is not suitable for applications at high temperatures. A previous study [17] suggested that the main reason is that the coating of most current optical fiber is a polymer that is sensitive to high temperatures. When the optical fiber is exposed to high temperatures or fire, the polymer coating easily softens. Finally, the optical properties and mechanical strength of the fiber may degrade due to damage [18,19]. Therefore, previous studies recommended coating fibers with a metal coating to develop high-temperature optical fibers [17,20,21]. However, the cost of such optical fibers is obviously much higher than fibers with ordinary polymer coating. Therefore, high-temperature coating approaches may not be suitable for civil engineering, which is price-sensitive. At present, only a few studies [22,23] have involved ordinary optical fibers and proposed calculation methods for temperature and strain coefficients. However, these studies were conducted under small strain levels and did not take large strain levels into account. Although previous studies [22,24] contributed a lot to the understanding of sensing properties of uncoated optical fiber at high temperatures, there is doubt whether those properties are suitable for practical conditions where optical fibers with coating are applied [23]. Therefore, it is still necessary to evaluate the sensing properties of ordinary optical fibers. If the temperature and strain coefficients of optical fiber at high temperature can be attained reliably and accurately, the method of embedding fiber optic sensors into FRP will solve the problem that the strain of FRP bars cannot be monitored at high temperature.

In this paper, the system of PPP-BOTDA was adopted. The ordinary single-mode optical fiber with UV resin (sensitive to light) was applied to the sensors. The highest temperature was up to 400 °C, because the highest temperature on the surface of FRP bars is around 400 °C when they are applied to concrete structures that are protected and exposed for less than 2 h in a fire [24]. The temperature and strain coefficients of the optical fiber in a high-temperature environment were measured and studied, especially considering the large strain levels. Finally, in this paper, a calculation method is proposed for temperature and strain coefficients of optical fiber at high temperatures. The experimental results reveal that the proposed coefficients are suitable for monitoring the temperature and strain of FRP bars at high temperatures.

## 2. Monitoring Principle of PPP-BOTDA

At present, the sensing mechanism of distributed optical fiber can be classified into Rayleigh scattering, Raman scattering, and Brillouin scattering. BOTDA technology is based on stimulated Brillouin backscattering. Brillouin backscattering will occur and an energy transfer will be generated between the two lasers as soon as the frequency difference between the two lasers introduced into the optical fiber from different ends is equal to the shift in the Brillouin center frequency (also called the Brillouin shift). The Brillouin shift of the optical fiber changes in proportion to the variation of strain or temperature along the fiber [25]. Therefore, this relationship is beneficial for measuring strain and temperature. From previous studies [26,27], the Brillouin shift, Δ*V_B_*, is linear with temperature change and strain change. The linear relationship can be given as follows:

(1) $$\Delta v_{B}\left(T,\varepsilon \right)=v_{B}-v_{B0}=C_{\varepsilon }\left(\varepsilon -\varepsilon_{0}\right)+C_{T}\left(T-T_{0}\right),$$

where *ε* is the strain (*με*); *T* is the temperature (°C); *V_B_* is the Brillouin frequency (MHz); *T_0_* and *ε_0_* are reference strain and temperature, respectively, corresponding to a reference Brillouin frequency, *V_B0_*; *C_ε_* is the strain coefficient (MHz/0.1%); and *C_T_* is the temperature coefficient (MHz/°C).

## 3. Temperature-Sensing Properties in the High-Temperature Field

Neubrex has developed an improved PPP-BOTDA system with better measurement accuracy in static measurement. This system has a spatial resolution of 10 cm, a strain resolution of 7.5 μm/m, and a temperature resolution of 0.75 °C, which are higher than previous products. In this test, Brillouin shifts were monitored with a Neubrescope (NBX-6050A, Neubrex Co., Ltd., Kobe, Japan).

In a previous study [16], the fiber strain coefficient (at normal temperature) and the temperature coefficient (20–60 °C) were tested and obtained. The results showed strain and temperature coefficients of 49.7 MHz/0.1% and 1.07 MHz/°C, respectively, which agreed well with the coefficients at ambient temperature. However, if exposed to a real fire, structures would be in a thermomechanical coupling environment and new optical fibers with coating would be applied only once. Therefore, it is necessary to consider optical fibers with coating and simulate the environment of both strain and high temperature when determining the two key coefficients.

### 3.1. Experimental Program

**Figure 1:**
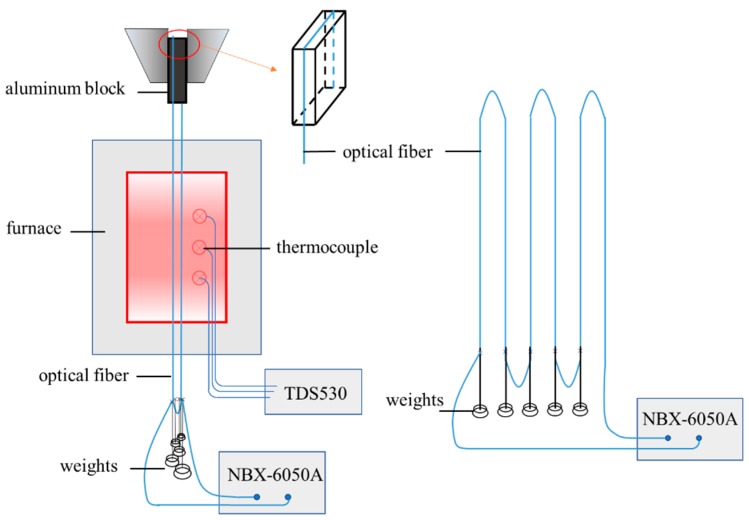
Schematic of experimental device.

**Figure 2:**
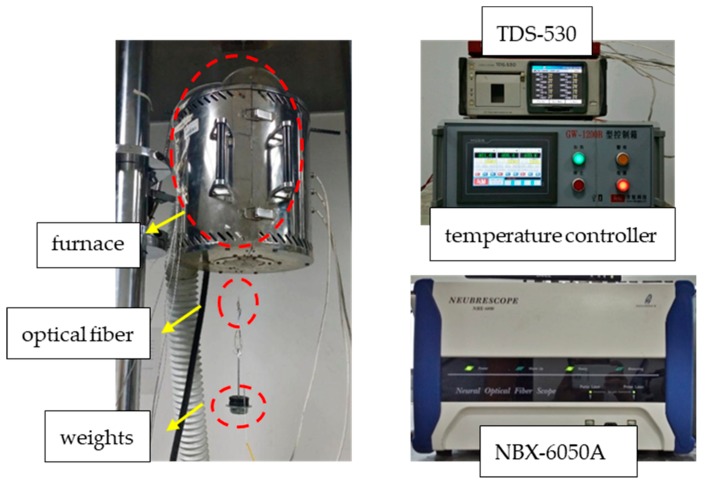
Details of test equipment.

Based on the relationships described by Formula (1), the optical fiber should be subjected to a certain initial strain before being exposed to high temperature. To acquire a more reliable conclusion, different initial strain levels were considered. Thus, a total of five bare optical fibers in series were tested, as shown in Figure 1. One of the five optical fibers was free; thus, its initial strain was 0 με. The other four initial strains were applied by hanging different weights at the ends of the optical fibers (Figure 2). Figure 2 shows the details of the test equipment. It was reported that the strain of optical fiber at room temperature could be reliably monitored by the optical fiber demodulator. The five measured initial strains were 0 με, 171 με, 424 με, 957 με, and 2107 με. These are all below 2500 με because that is the critical value for steel yields in basalt fiber reinforced polymer (BFRP) strengthening beams by the near surface mounted (NSM) technique, and it is consistent with previous studies [20,22].

In the past, few studies have considered large initial strains. One study [22] considered six initial strain levels. However, the maximum initial strain was 1639 με, which is far lower than that in engineering applications. In addition, the temperature coefficient might be different if the initial strain is very large. Therefore, it is necessary to consider larger strain ranges, especially considering that, when structures reach the ultimate limit state after cracking, optical fibers embedded in FRP bars will reach a relatively large strain, close to 10,000 με. An extra temperature coefficient experiment under large initial strain levels (in the range of 2,500–9,000 με) was added. The measured initial strain values were 3154 με, 4693 με, 5873 με, 7328 με, 8149 με, and 9140 με. Then, the fibers were heated from room temperature to 400 °C. At every 30 °C, the temperature and the Brillouin frequency shifts were collected and recorded by a data logger (model number TDS-530) and an optical fiber demodulator (model number NBX-6050A), respectively. After the target temperature was reached and the heating process was complete, the furnace was naturally cooled to room temperature. After that, the same heating process was performed again. Finally, the heating process was repeated a total of five times.

### 3.2. Experimental Results

#### 3.2.1. Initial Strain Levels in the Range of 0–2500 με

**Figure 3:**
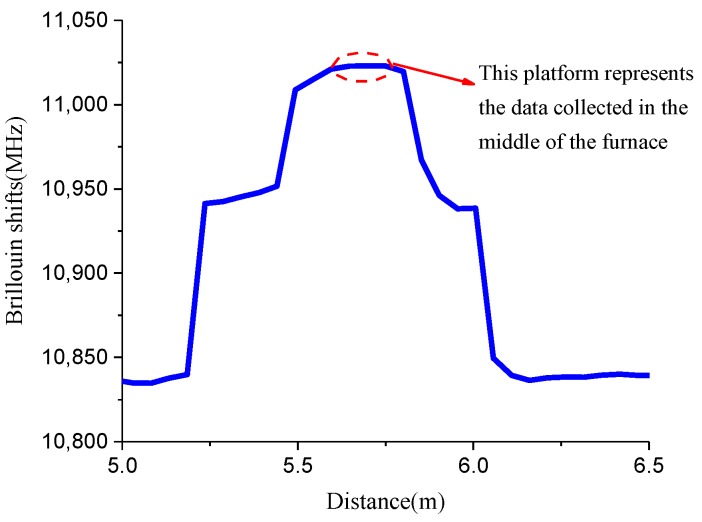
Typical plot collected by NBX-6050A in temperature coefficient test.

There were five strain levels in each heating process. Figure 3 shows the relationship between Brillouin shift and distance, which is a typical plot collected by the demodulator. Then, the platform data from the figure were processed through MATLAB to obtain the Brillouin shifts at the desired location of an optical fiber. From the previous studies, there are two methods to analyze the relationship between Brillouin shift and temperature. One method is to linearly fit the experimental data under different temperature ranges, and the other method is to consider that the Brillouin shift is quadratic with temperature. In this case, the first method was applied by dividing the whole heating process into different temperature ranges. Then, the temperature coefficients under the different temperature ranges can be attained by linearly fitting the experimental data. This method seems to be extremely simple but has low accuracy, especially when the temperature is over 200 °C. So, the second method is mainly discussed in the following section.

**Figure 4:**
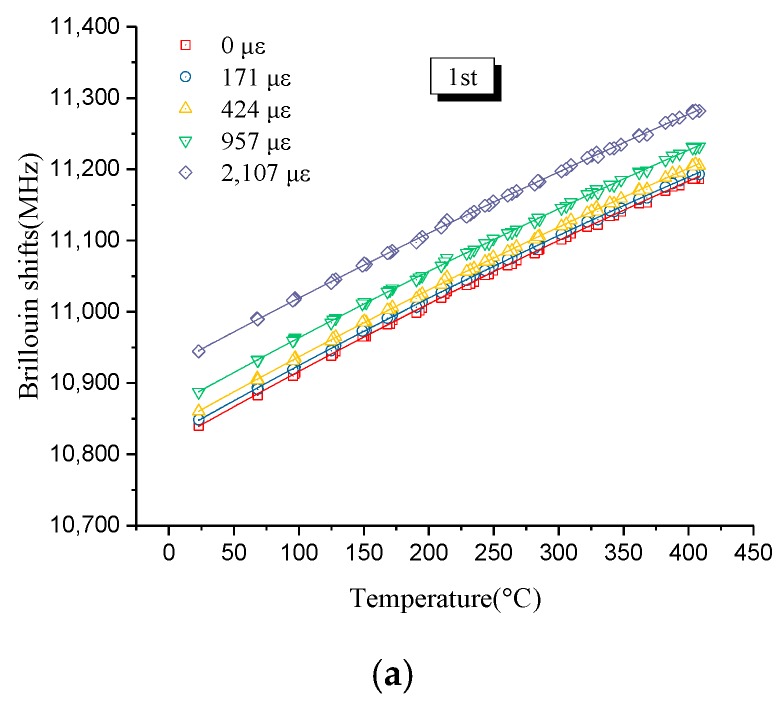

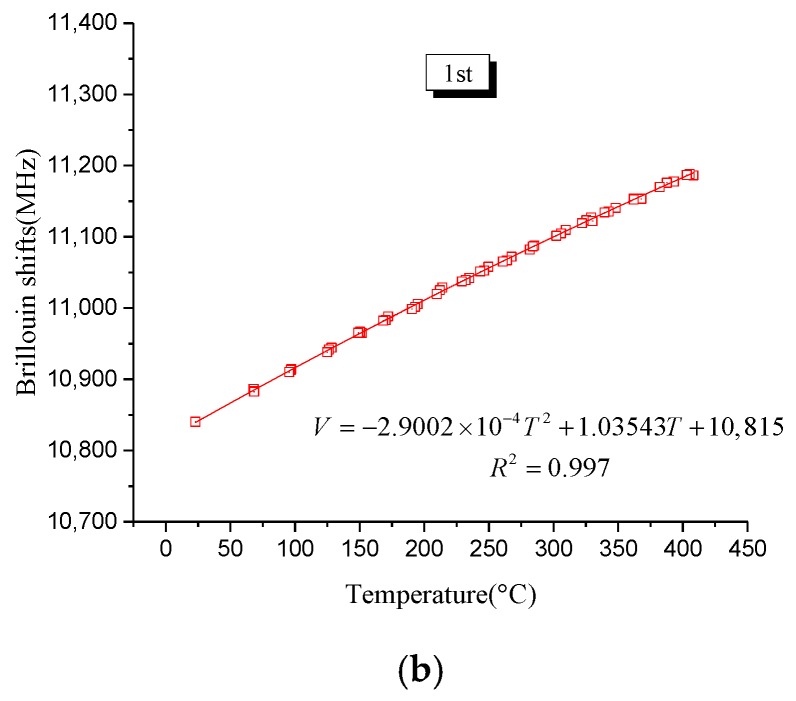
Brillouin shifts versus temperature: (**a**) At different strain levels and (**b**) regression analysis at zero strain.

A previous study [22] noted that Brillouin shifts were quadratic with temperature and the Brillouin shift–temperature curves at various initial strains were approximately parallel. Figure 4a also shows the quadratic relations between Brillouin shifts and temperature at different initial strains in the first heating process. The particular curve at zero strain in Figure 4a was chosen to be fit using a parabolic equation, as represented in Figure 4b. The parabolic regression equation accurately fit the test data, as indicated by the coefficient of correlation, which is 0.9997. As such, the regression equation can be written as follows:

(2) Δv=(αT+β)(T−20).

In other words, the temperature sensitivity coefficient can be expressed as follows:

(3) $$C_{T}=\alpha T+\beta ,$$

where *α* equals −2.9 × 10^−4^ and *β* equals 1.035 from Figure 4b.

Similarly, the temperature sensitivity coefficient under other initial strains can be expressed as above. The detailed values of α and *β* are shown in Table 1. In a previous study [22], only the values of *α* and *β* at zero strain were attained, and they were −3.464 × 10^−4^ and 1.11, respectively. Then, because of the parallel relations of curves at different initial strains, the values of *α* and *β* at other initial strains were considered to be the same, as those at zero strain and were ignored. However, the values of *α* and *β* are different in the range of 0–2500 με, from Table 1, which means the parallel relations proposed in Reference [22] are not reasonable, because optical fibers with coating were applied in this case. From Table 1, different initial strains may have an effect on temperature coefficients and Formula (3) may need to take different initial strains into consideration.

#### 3.2.2. Initial Strain in the Range of 2500–9000 με

**Figure 5:**
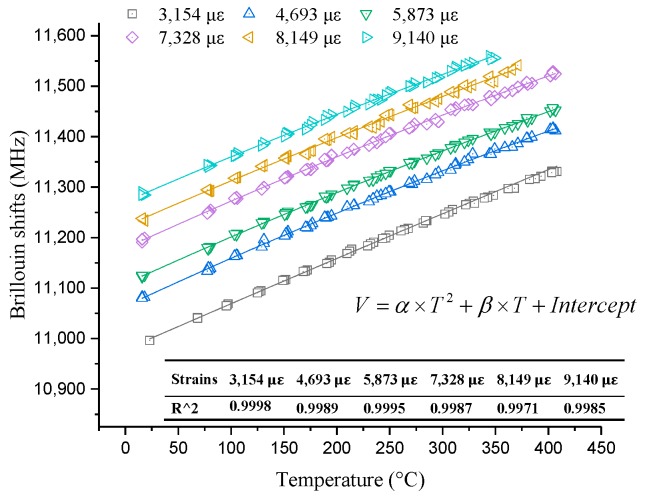
Experimental results at large strains.

**Figure 6:**
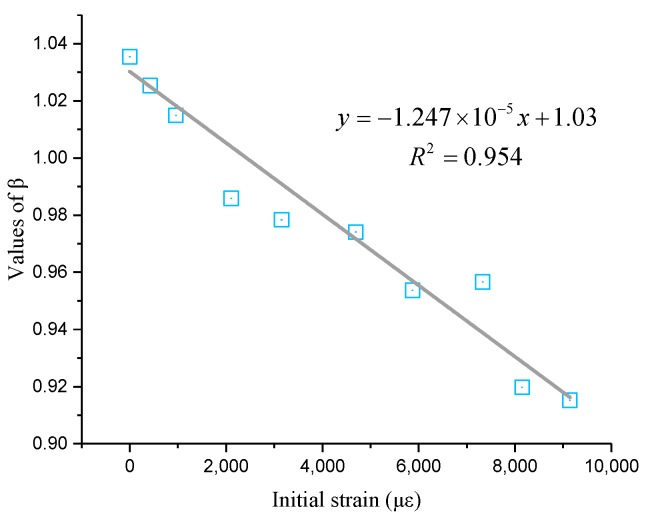
Linear fitting result of β and strain.

Figure 5 shows the quadratic relationship between Brillouin shifts and temperature when the initial strain is in the range of 2500–9000 με. The fitting results are great because six correlation coefficients are larger than 0.99. Therefore, six temperature coefficient formulas can be derived from the corresponding quadratic curves. Similarly, the temperature coefficient formulas are decided by *α* and *β*, as shown in Figure 5. The values of *α* and *β* shown in Table 2 have slight fluctuations. Therefore, the initial strains do have an effect on the temperature coefficient. The values of *β* decrease with the increasing initial strains from Table 1 and Table 2. However, the values of *α* do not show obvious regularity with increasing strain. Therefore, in order to conclude a general temperature coefficient formula, the values of *α* are averaged after maximum and minimum values are removed. The detailed average value of *α* is −2.64 × 10^−4^. In addition, from Figure 6, it seems the relationship between *β* and strain is linear. The linear fitting result is shown in Figure 6. Thus, the relationship between *β* and initial strain can be expressed as follows:

(4) $$\beta =-1.247\times 10^{-5}\varepsilon +1.03.$$

Furthermore, the temperature sensitivity coefficient can be expressed as follows:

(5) $$C_{T}=-2.64\times 10^{-4}T-1.247\times 10^{-5}\varepsilon +1.03.$$

Both temperature and strain are taken into considerations in Formula (5), which is suitable for all different strains and is more reasonable.

#### 3.2.3. Analysis of Accuracy

Although the above section discusses the Brillouin shift–temperature relationship in detail and the results seem to be reasonable, temperature-sensing accuracy is another important parameter to evaluate the sensing properties of optical fibers. During the experiments, the temperatures collected by thermocouples connected to the data logger were accurate and considered as real temperatures. The Brillouin shifts recorded by the demodulator can be used to calculate the temperature according to Formula (1), where the above-measured temperature coefficient, Formula (5), was applied.

**Figure 7:**
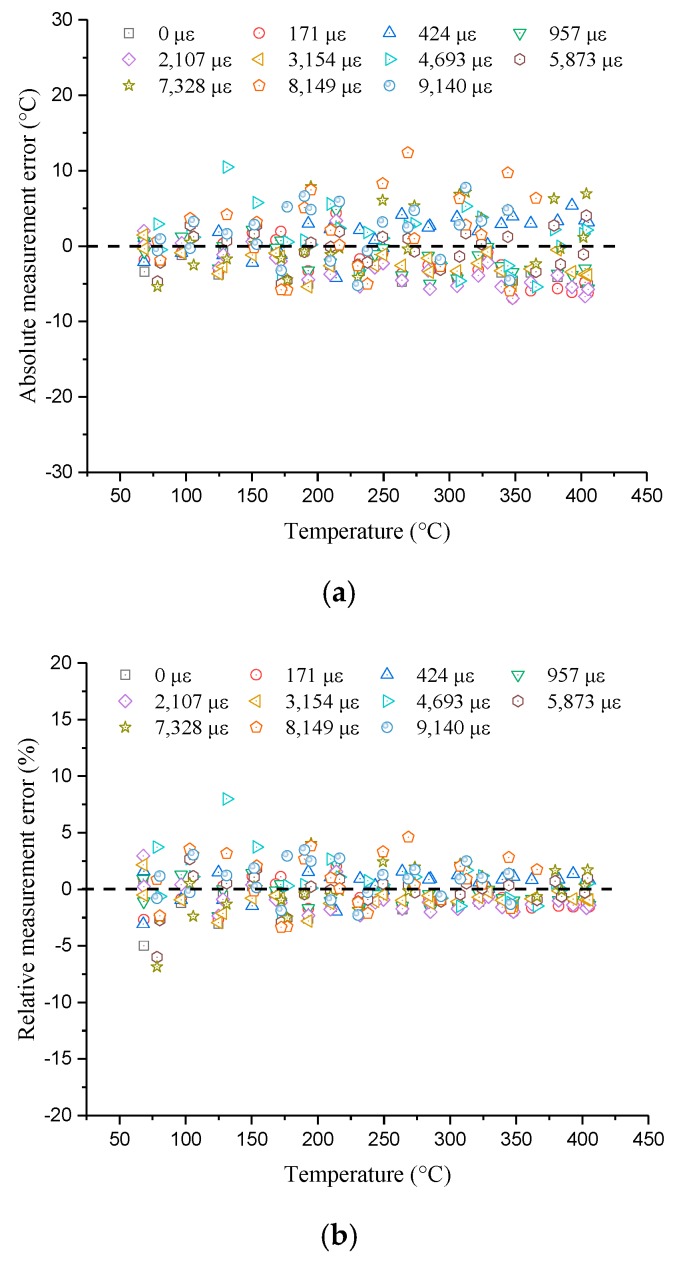
Measurement errors (using temperature coefficient formula): (**a**) Absolute values; (**b**) relative values.

So, Figure 7 compares temperatures measured by the optical fiber demodulator and real temperatures by showing the measurement errors, including absolute and relative errors. The relative values mean the absolute values divided by the corresponding real temperature. From Figure 7a, most of the absolute values are within the range from −8 °C to 8 °C and float around zero. Meanwhile, most of the relative errors range from −5% to 5% from Figure 7b, indicating that measurement errors are very small. Obviously, the quadratic relationship between Brillouin shifts and temperature has high accuracy. Therefore, it can be concluded that temperature coefficients decrease as temperature increases and Formula (5) is suitable for the strain range of 0–9000 με because different initial strains and high temperatures are taken into consideration.

### 3.3. Discussion 

Although the above section shows great results, the effect of coating and the reason why temperature coefficients will decrease at high temperatures still need to be discussed. It is common sense that the coating will have an effect on the properties of optical fiber. The coating of optical fiber is so hard to strip off that any further stress imposed on the bare optical fiber would break it. Thus, this section analyzes the coating effect by comparing the results attained from multiple heating processes, as in a previous study [17]. 

**Figure 8:**
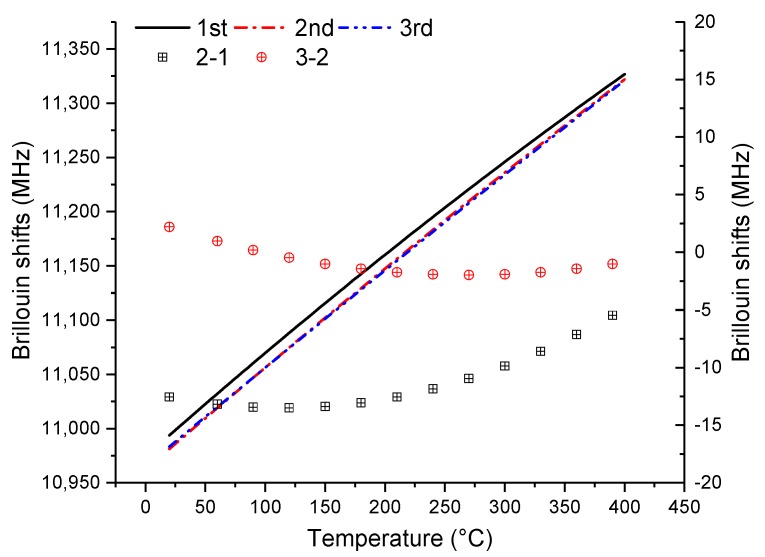
Influence of coating burning on optical fiber at 3154 με.

Figure 8 shows three heating results at 3154 με. The black, red, and green lines represent the first, second, and third heating results, respectively. The square black points represent the Brillouin shift difference between the first and second measurements, and the circular red points represent the Brillouin shift difference between the second and third measurements. The Brillouin shifts of the second measurement are between 5 and 13.5 MHz lower than those of the first measurement, indicating that the coating would introduce tensile stress when the fiber was drawing [17]. The difference becomes smaller with temperature; especially when the temperature is up to 400 °C, the difference between the two measurements is 5 MHz, which is allowable. This is because the coating burning would occur around 350 °C, which would cause coating pyrolysis and further introduce additional compressive stress, counteracting the tensile stress of fiber drawing [17]. Furthermore, the maximum difference between the first and second results is around 13.5 MHz, which results from the strain changes caused by the coating. From Reference [22], the strain coefficient can be taken as 47.9 MHz/0.1% when the maximum extra strain occurs. Therefore, the maximum extra strain caused by the coating is around 280 με, according to Formula (1). The results are the same under other initial strains. Thus, experimental errors caused by this extra strain can be ignored when the initial strains are very large; otherwise, they cannot be ignored. Meanwhile, this extra strain can be eliminated with temperature. Additionally, the difference between the second and third measurements is almost close to zero, indicating that the coating may be burned out after the first heating process. 

**Figure 9:**
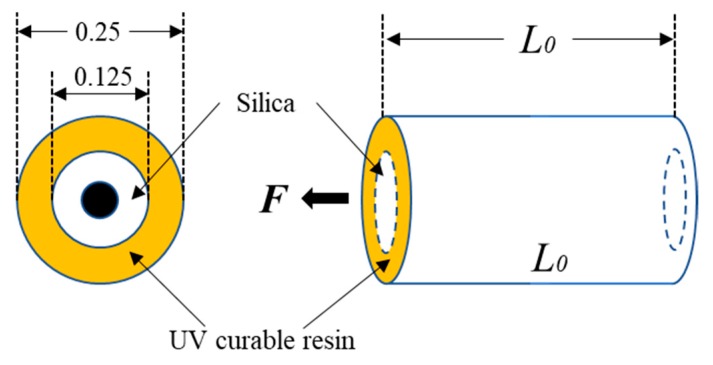
Force model applied to analyze the relationship between strain and temperature.

The temperature coefficient is mainly applied to represent the temperature sensing of optical fibers. It can be understood as the difference in Brillouin shifts per unit of temperature, according to Formula (1). The above section confirms that the relationship is nonlinear instead of linear at high temperature, indicating that the temperature coefficient decreases as the temperature rises. This can be attributed to the velocity variations of the acoustic wave [28] or the nonlinear relationship between the modulus of the silica materials and temperature [29,30]. To further explain the reason for a decrease in temperature coefficients, a simplified force model is shown in Figure 9. Except for the non-uniform temperature field in the furnace, length L_0_ means the length of the optical fiber in the furnace. The total elongation, ΔL, of this part is equal to the following:

(6) $$\Delta L=\varepsilon (T)L_{0}=\frac{FL_{0}}{E(T)A(T)},$$

where ε(T) is the strain; F is the force, which is caused by weights; and E(T) and A(T) are the equivalent Young’s modulus and cross-sectional area of the optical fiber, respectively. Neglecting the relative displacement between silica and coating, the tensile rigidity, E(T)A(T), can be written as follows:

(7) $$E(T)A(T)=E_{s}(T)A_{s}(T)+E_{u}(T)A_{u}(T),$$

where $E_{s}(T)$ and $A_{s}(T)$ are Young’s modulus and the cross-sectional area of silica, respectively, and $E_{u}(T)$ and $A_{u}(T)$ are Young’s modulus and the cross-sectional area of the coating (UV curable resin), respectively. From Reference [29], Young’s modulus of silica will increase nonlinearly from 72 GPa to 82 GPa when the temperature increases from 20 °C to 1400 °C. The area of silica can be determined by using a diameter of 0.125 mm and a thermal expansion of 5.5 × 10^−7^/°C. The Young’s modulus of UV curable resin is around 2 GPa at ambient temperature [31]. The coating will soften and, further, the total volume will decrease with temperature. Therefore, Young’s modulus and the area of coating must decrease with temperature. However, there is no detailed relationship that can describe this trend. 

The strain that is a function of temperature can be calculated by using the following:

(8) $$\varepsilon (T)=\frac{F}{E_{s}(T)A_{s}(T)+E_{u}(T)A_{u}(T)}.$$

It is estimated that the increase of $E_{s}(T)A_{s}(T)$ is larger than the decrease of $E_{u}(T)A_{u}(T)$ at high temperature, because Young’s modulus of the silica is much larger than that of the coating. Thus, E(T)A(T) will increase, which further causes strain reduction with temperature. The reduction in strain will lead to a Brillouin shift reduction, according to Formula (1), which can account for the nonlinear relationship between Brillouin shifts and temperature. Unfortunately, many properties of silica and UV curable resin are not given by the manufacturer. Thus, only qualitative analysis can be discussed in this case.

Furthermore, other factors, such as the dopant diffusion of the core and fiber devitrification, will also increase the loss of the optical fiber when the temperature is up to 1200 °C, which was not taken into consideration here [17].

## 4. Strain-Sensing Properties in the High-Temperature Field

### 4.1. Experimental Program

The above paragraphs describe the temperature coefficient of optical fibers, which decides the value of $C_{T}$ in Formula (1). However, optical fiber strain sensing is another important property in thermomechanical coupling conditions. The study of the relationship between the Brillouin shift and strain was carried out for two purposes. The first purpose was to study whether the tested strain coefficient was consistent with the value at ambient temperature. The other purpose was to acquire an accurate strain coefficient suitable for thermomechanical coupling. 

**Figure 10:**
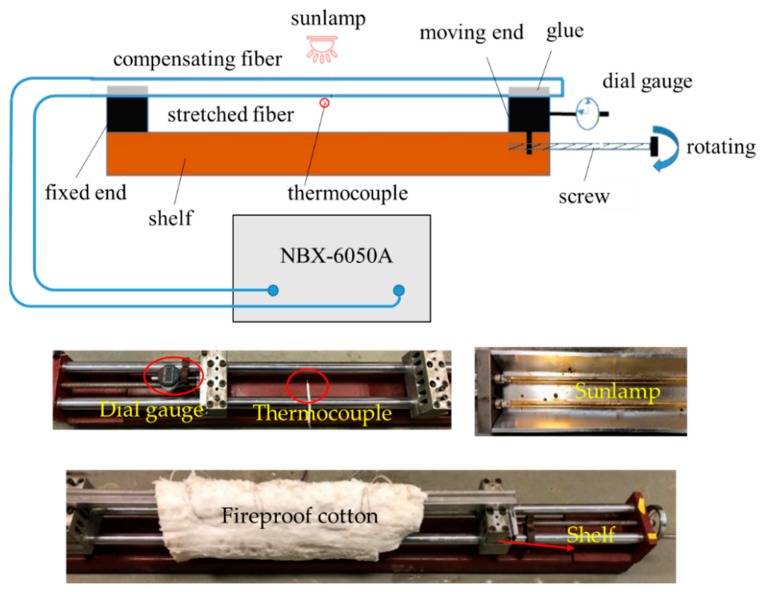
Test device for strain coefficient of optical fiber.

The experimental setup for optical fibers is shown in Figure 10. Two optical fibers were used, as follows: One was bare and used as stretched fiber bonded on the fixed end with the moving end through the glue, and the other was free and used as compensated fiber in series with the stretched fiber. To obtain a stable high-temperature condition simultaneously, a sunlamp was placed beside a shelf wrapped in fireproof cotton. Different temperatures were achieved by adjusting the distance between the fiber and sunlamp. The moving end was shifted by rotating the screw bar along the shelf. During the experiments, the distance change that was easily transformed into the optical fiber strain could be measured by a displacement transducer. Therefore, the tensile strain was exactly controlled at each step by controlling the moving distance of the end. The Brillouin shifts along the optical fiber were measured by the optical fiber demodulator, and the temperature was collected by a data logger at the same time.

The experiments were repeated multiple times. Additionally, considering the ultimate strain of BFRP bars in ambient temperature (approximately 20,000 με) and the bonding performance of resin, the experiments were divided into two working conditions, small strain (0–2500 με) and large strain (2500–17,500 με), because 2500 με is a critical value, as mentioned above. The small strain was applied to simulate the state before the yielding of steel bars or after the appearance of apparent damage, and the large strain was applied to simulate the state where the steel bars might yield while the BFRP bars still worked or after serious damage to the structure occurred. Due to limited experimental conditions, the stable achieved temperatures were 100 °C and 350 °C, respectively.

### 4.2. Results

**Figure 11:**
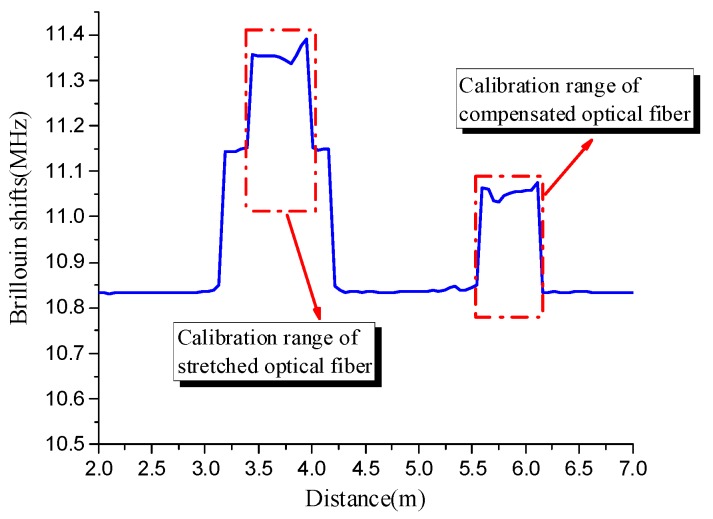
Typical plot collected by NBX-6050A in the strain coefficient test.

Figure 11 shows the typical Brillouin shifts collected by the optical fiber demodulator in the strain coefficient experiments. The Brillouin shift changes in the gauge length of the stretched and compensating fibers are given in Figure 11. The Brillouin shift of stretched fiber minus the compensating fiber equals the Brillouin shift caused by strain. Theoretically, the Brillouin shift of optical fiber in the calibration range is a fixed value that can be directly applied. However, the results of Figure 11 do not match the theory. The Brillouin shift curve fluctuates because the temperature is not uniform. 

**Figure 12:**
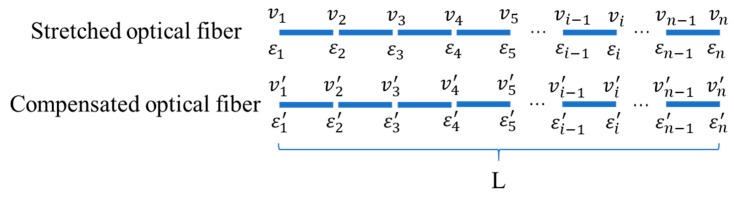
Schematic view of data processing.

Therefore, the Brillouin shift data are processed according to the following procedures:

First, as shown in Figure 12, the fiber is divided into n segments. The total length of the fiber is L, and the length of each segment is l. Then, the Brillouin shifts of the *i*th segment of the stretched optical fiber can be expressed as follows:

(9) $$v_{i}=C_{\varepsilon }\left(\varepsilon_{i}-\varepsilon_{0}\right)+C_{T}\left(T_{i}-T_{0}\right).$$

Similarly, the corresponding ith segment of the stretched optical fiber can be written as follows:

(10) $$v_{i}^{\prime }=C_{\varepsilon }\left(\varepsilon_{i}^{\prime }-\varepsilon_{0}\right)+C_{T}\left(T_{i}^{\prime }-T_{0}\right).$$

Second, the difference in Brillouin shifts between the stretched fiber and the compensated fiber can be calculated by subtracting Formula (9) and Formula (10), as follows:

(11) $$v_{i}-v_{i}^{\prime }=C_{\varepsilon }\left(\varepsilon_{i}-\varepsilon_{i}^{\prime }\right)+C_{T}\left(T_{i}-T_{i}^{\prime }\right).$$

It is noticeable that the temperatures of the stretched and compensated fiber are the same at the ith segment. The left side of Formula (11) can be expressed as $\bar{v_{i}}$ for simplicity, then the formula can be further simplified as follows:

(12) $${\bar{v}}_{i}=C_{\varepsilon }\left(\varepsilon_{i}-\varepsilon_{i}^{\prime }\right).$$

Third, after both sides of Formula (12) are multiplied by *l* except the first and last formulas are multiplied by *l*/2, it can be expanded as follows:

(13) $$\{\begin{array}{c} {\bar{v}}_{1}\frac{l}{2}=C_{\varepsilon }\left(\varepsilon_{1}-\varepsilon_{1}^{\prime }\right)\frac{l}{2}\\ {\bar{v}}_{2}l=C_{\varepsilon }\left(\varepsilon_{2}-\varepsilon_{2}^{\prime }\right)l\\ {\bar{v}}_{3}l=C_{\varepsilon }\left(\varepsilon_{3}-\varepsilon_{3}^{\prime }\right)l\\ \ldots \\ {\bar{v}}_{i}l=C_{\varepsilon }\left(\varepsilon_{i}-\varepsilon_{i}^{\prime }\right)l\\ \ldots \\ {\bar{v}}_{n}\frac{l}{2}=C_{\varepsilon }\left(\varepsilon_{n}-\varepsilon_{n}^{\prime }\right)\frac{l}{2} \end{array}$$

Then, adding both sides of each equation in Formula (13), the new formula can be written as follows:

(14) $${\bar{v}}_{1}\frac{l}{2}+{\bar{v}}_{2}l+{\bar{v}}_{3}l+\ldots +{\bar{v}}_{i}l+\ldots +{\bar{v}}_{n}\frac{l}{2}=C_{\varepsilon }\{\left(\varepsilon_{1}-\varepsilon_{1}^{\prime }\right)\frac{l}{2}+\left(\varepsilon_{2}-\varepsilon_{2}^{\prime }\right)l+\left(\varepsilon_{3}-\varepsilon_{3}^{\prime }\right)l+\ldots +\left(\varepsilon_{i}-\varepsilon_{i}^{\prime }\right)l+\ldots +\left(\varepsilon_{n}-\varepsilon_{n}^{\prime }\right)\frac{l}{2}\}$$

The strain measured by the dial gauge is expressed as $\varepsilon_{av}$. Obviously, the left side of Formula (14) indicates the real elongation of the optical fiber, which is $\varepsilon_{av}L$. If n→∞ or l→0, Formula (14) can be simplified as follows:

(15) $${\bar{v}}_{1}\frac{l}{2}+{\bar{v}}_{2}l+{\bar{v}}_{3}l+\ldots +{\bar{v}}_{i}l+\ldots +{\bar{v}}_{n}\frac{l}{2}=C_{\varepsilon }\varepsilon_{av}L$$

Finally, the strain coefficient, *C_ε_*, can be calculated according to Formula (15). The above analysis can be programmed by MATLAB and the strain coefficients can be calculated and obtained automatically.

**Figure 13:**
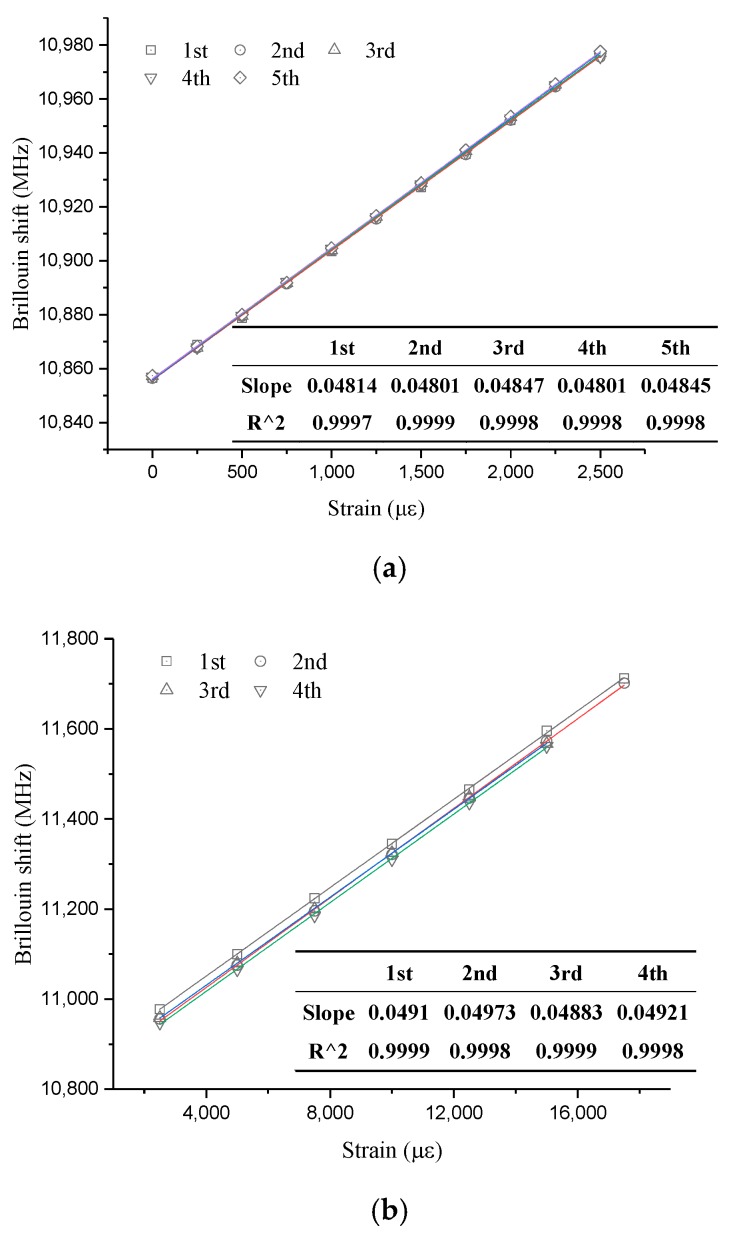
Experimental results for strain sensing at ambient temperature: (**a**) Small strain; (**b**) large strain.

**Figure 14:**
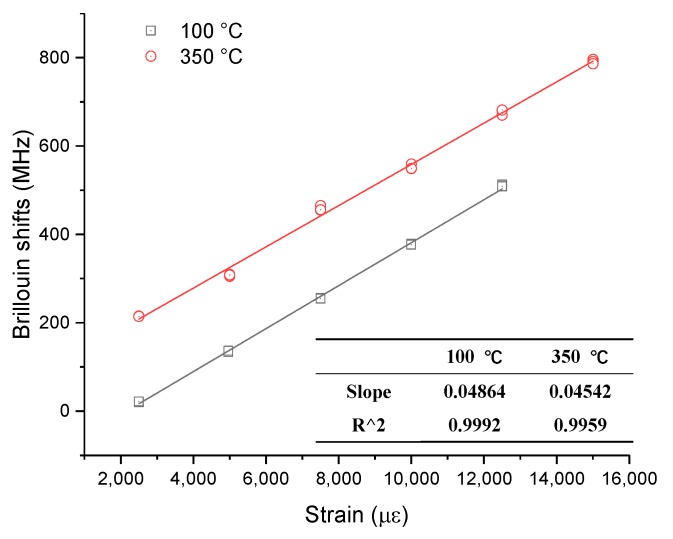
Experimental results for strain sensing at 100 °C and 350 °C.

As a consequence of the limited experimental conditions, the tested values only included strain coefficients at ambient temperature, 100 °C, and 350 °C. Figure 13 and Figure 14 show the relationship between Brillouin shift and strain under three temperature levels. It is obvious that all the correlation coefficients are larger than 0.99, which also indicates great strain-sensing linearity, regardless of room or high-temperature conditions. The slope of each fitting line in these figures is the value of the corresponding strain coefficient. The specific results are listed in the two figures. 

From Figure 13, it is obvious that the strain-sensing repeatability for optical fibers is great because the results of five tests under small strains are the same and those of four tests under large strains are almost equal. On average, strain coefficients under small strain and large strain at ambient temperature are 48.24 MHz/0.1% and 49.25 MHz/0.1%, respectively. The values are very close to the value at ambient temperature, which is 49.7 MHz/0.1%. Therefore, it confirms that the value at ambient temperature is 49.7 MHz/0.1%, again.

A previous study [20] analyzed the strain sensing of optical fibers at different high temperatures and described a strain coefficient formula when the temperature is below 700 °C. However, the maximum strain in that study was around 3000 με, indicating that the larger strain working level still needs to be considered. Therefore, Figure 14 shows only one result at two high temperatures under large strains because of the great strain-sensing repeatability in previous tests. From Figure 14, the strain coefficients at 100 °C and 350 °C are 48.64 MHz/0.1% and 45.42 MHz/0.1%, respectively, which are close to the coefficients measured under small strains in Reference [20]. 

### 4.3. Discussion

**Figure 15:**
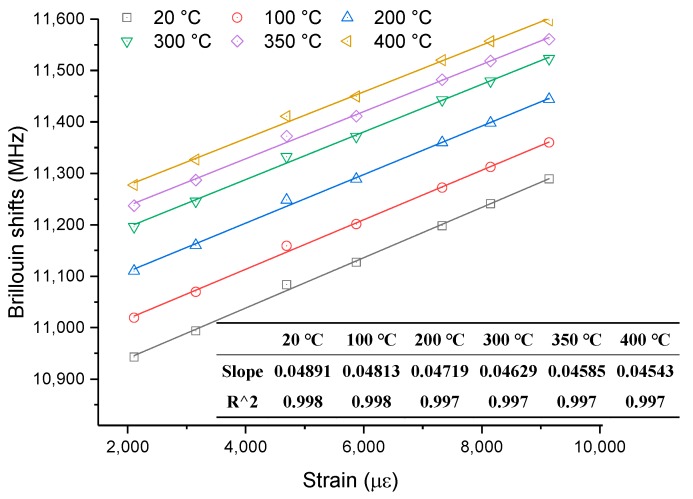
Theoretical relationships of strain sensing at different temperatures.

As only two high-temperature conditions were simulated in this condition, it is difficult to further analyze the results from the thermal or mechanical properties of the optical fibers. Fortunately, the above section about temperature sensing of optical fibers considers a thermomechanical coupling condition including six large initial strains that can be applied to verify the reliability of strain-sensing measurements and compensate for the limited high-temperature conditions. The detailed method transforms Figure 5 into Figure 15, which shows the theoretical relationships between Brillouin shifts and strain at different high temperatures. The maximum temperature is 400 °C, which is also the maximum temperature in Figure 5. 

From Figure 15, it can be observed that the Brillouin shifts linearly increase with strain at different temperatures. The slope of each Brillouin shift–strain curve (strain coefficient) at each temperature can be determined, and these are shown in Figure 15. The strain coefficients are 49.25 MHz/0.1%, 48.13 MHz/0.1%, and 45.85 MHz/0.1%, corresponding to temperatures of 20 °C, 100 °C, and 350 °C, respectively. Those two coefficients are very close to the above experimental results, which are 48.91 MHz/0.1%, 48.64 MHz/0.1%, and 45.42 MHz/0.1%, respectively. Therefore, it confirms that the experimental results for temperature sensing and strain sensing of the optical fibers are reliable. 

**Figure 16:**
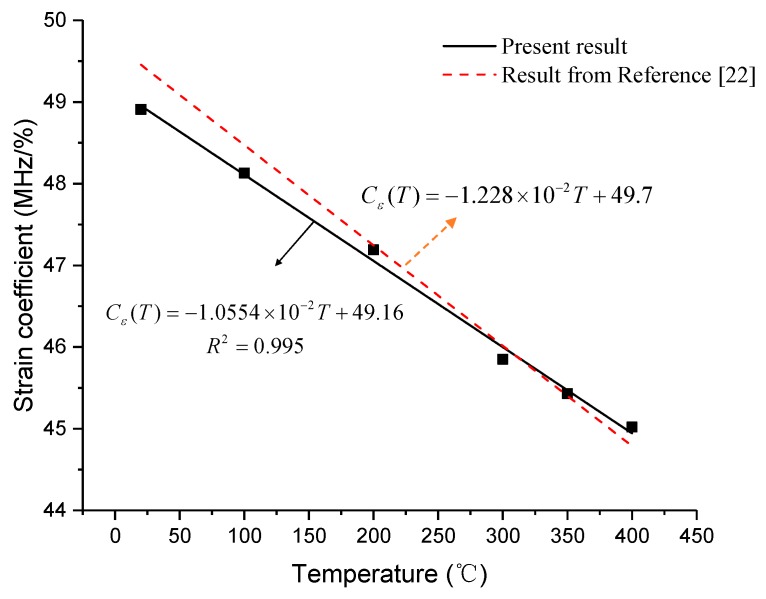
Temperature dependence of strain coefficient.

It is worth noting from Figure 16 that the strain coefficient will decrease as temperature rises. The black line represents the strain coefficient as a function of temperature for six points. The linear formula of strain coefficients can be expressed as

(16) $$C_{\varepsilon }\;=\;-1.0554\times 10^{-2}T\;+\;49.16$$

The dotted red line represents the relationship between strain coefficient and temperature, attained from Reference [22]. The largest difference of strain coefficient between the two lines is 0.544 MHz/0.1% at ambient temperature, which may be due to the effect of the coating. However, this difference is so small that it can be ignored. So, the strain coefficient result from Figure 16 is consistent with the results from References [20,22], which further indicates that larger strain conditions will not influence the strain coefficient. 

As for the reason for the decrease in the strain coefficient with temperature, it may be due to the increase in Young’s modulus of silica with temperature, as reported in a previous study [20]. However, this explanation still needs further verification with more experiments. 

## 5. Conclusions

The sensing properties of ordinary single-mode optical fibers were mainly studied at high temperature, because this kind of optical fiber with coating is often directly applied in practical conditions. The most important contribution is the calibration of two key coefficients, temperature and strain coefficients, in a thermomechanical coupling condition with large strain conditions, which have not been taken into account by previous studies. The following conclusions and remarks can be made:

(1) Although burning of the coating will have an effect on sensing properties, ordinary optical fibers are still valid because the nonlinear relationship between Brillouin shift and temperature is great at different initial strains. Similarly, the linear relationship between Brillouin shift and strain is also great at different temperatures.
(2) The temperature and strain coefficients tend to decrease with temperature, mainly because of the increase in Young’s modulus of silica with temperature, which will further change the strain of optical fibers.
(3) The temperature coefficient can be determined by Formula (5), which is suitable for the range of 0–9000 με. The strain coefficient can be determined by Formula (16), which is consistent with experimental results and previous studies.
(4) The research and development of smart FRP bars may solve the problem of obtaining strain at high temperatures. The calibration of two key parameters (temperature and strain coefficients) under different levels guarantees the reliability of the subsequent measured data. This result provides a basis for further high-temperature studies of smart FRP bars.

## Figures and Tables

**Table 1:** Values of α and β at different initial strains (below 2500 με).

	0 με	171 με	424 με	957 με	2107 με
*α*	−2.9 × 10^−4^	−3.1 × 10^−4^	−2.86 × 10^−4^	−2.73 × 10^−4^	−2.51 × 10^−4^
*β*	1.035	1.037	1.025	1.015	0.986

**Table 2:** Values of *α* and *β* at different initial strains (above 2500 με).

	3154 με	4693 με	5873 με	7328 με	8149 με	9140 με
*α*	−2.43 × 10^−4^	−2.68 × 10^−4^	−2.52 × 10^−4^	−2.61 × 10^−4^	−2.11 × 10^−4^	−2.49 × 10^−4^
*β*	0.978	0.974	0.954	0.957	0.920	0.915

## References

[1] Barrias A., Casas J.R., Villalba S. (2018). Embedded Distributed Optical Fiber Sensors in Reinforced Concrete Structures-A Case Study. Sensors.

[2] Lee J.R., Ryu C.Y., Koo B.Y., Kang S.G., Hong C.S., Kim C.G. (2003). In-flight health monitoring of a subscale wing using a fiber Bragg grating sensor system. Smart Mater. Struct..

[3] Baldwin C., Poloso T., Chen P., Niemczuk J., Kiddy J., Ealy C., Liu S.C. (2001). Structural monitoring of composite marine piles using fiber optic sensors. Smart Structures and Materials 2001: Smart Systems for Bridges, Structures, and Highways.

[4] Zhang Y.X., Shi Y.F., Zhao Y.D., Fu L.R., Yang J.S. (2017). Determining the Cause of Damages in a Multiarch Tunnel Structure through Field Investigation and Numerical Analysis. J. Perform. Constr. Facil..

[5] Chong K.P., Carino N.J., Washer G. (2003). Health monitoring of civil infrastructures. Smart Mater. Struct..

[6] Zhang Y.X., Liu C., Lu W.H., Xie H.B., Peng H. (2019). Comparative Study of RC Members with Strengthening Using Strain Hardening Cementitious Composite and Fiber Reinforced Mortar. J. Test. Eval..

[7] Wang G., Pran K., Sagvolden G., Havsgard G.B., Jensen A.E., Johnson G.A., Vohra S.T. (2001). Ship hull structure monitoring using fibre optic sensors. Smart Mater. Struct..

[8] Ohno H., Naruse H., Kihara M., Shimada A. (2001). Invited paper—Industrial applications of the BOTDR optical fiber strain sensor. Opt. Fiber Technol..

[9] Wolfbeis O.S. (2008). Fiber-optic chemical sensors and biosensors. Anal. Chem..

[10] Kinet D., Megret P., Goossen K.W., Qiu L., Heider D., Caucheteur C. (2014). Fiber Bragg Grating Sensors toward Structural Health Monitoring in Composite Materials: Challenges and Solutions. Sensors.

[11] Cho S.B., Lee J.J., Kwon I.B. (2006). Pulse base effect on the strain measurement of a Brillouin-scattering-based distributed optical fiber sensor. Smart Mater. Struct..

[12] Yang C.Q., Wu Z.S., Ye L.P. (2006). Self-diagnosis of hybrid CFRP rods and as-strengthened concrete structures. J. Intell. Mater. Syst. Struct..

[13] Tang Y., Wu Z., Yang C., Shen S., Wu G., Hong W. Development of self-sensing BFRP bars with distributed optic fiber sensors. Proceedings of the Smart Sensor Phenomena, Technology, Networks, and Systems 2009.

[14] Wu Z.S., Zhang H., Yang C.Q. (2010). Development and Performance Evaluation of Non-slippage Optical Fiber as Brillouin Scattering-based Distributed Sensors. Struct. Health Monit..

[15] Tang Y., Wu Z. (2016). Distributed Long-Gauge Optical Fiber Sensors Based Self-Sensing FRP Bar for Concrete Structure. Sensors.

[16] Tang Y.S., Wu Z.S., Yang C.Q., Wu G., Shen S. (2010). A new type of smart basalt fiber-reinforced polymer bars as both reinforcements and sensors for civil engineering application. Smart Mater. Struct..

[17] Xu P.B., Dong Y.K., Zhou D.W., Fu C., Zhang J.W., Zhang H.Y., Lu Z.W., Chen L., Bao X.Y. (2016). 1200 degrees C high-temperature distributed optical fiber sensing using Brillouin optical time domain analysis. Appl. Opt..

[18] Kurkjian C.R., Krause J.T., Matthewson M.J. (1989). Strength and fatigue of silica optical fibers. J. Lightwave Technol..

[19] Rose A.H. (1997). Devitrification in annealed optical fiber. J. Lightwave Technol..

[20] Xu P.B., Ba D.X., He W.M., Hu H.P., Dong Y.K. (2018). Distributed Brillouin optical fiber temperature and strain sensing at a high temperature up to 1000 degrees C by using an annealed gold-coated fiber. Opt. Express.

[21] Ruiz-Lombera R., Laarossi I., Rodriguez-Cobo L., Quintela M.A., Lopez-Higuera J.M., Mirapeix J. (2017). Distributed High-Temperature Optical Fiber Sensor Based on a Brillouin Optical Time Domain Analyzer and Multimode Gold-Coated Fiber. IEEE Sens. J..

[22] Bao Y., Chen G.D. (2016). Temperature-dependent strain and temperature sensitivities of fused silica single mode fiber sensors with pulse pre-pump Brillouin optical time domain analysis. Meas. Sci. Technol..

[23] Bao Y., Hoehler M.S., Smith C.M., Bundy M., Chen G.D. (2017). Temperature measurement and damage detection in concrete beams exposed to fire using PPP-BOTDA based fiber optic sensors. Smart Mater. Struct..

[24] Firmo J.P., Correia J.R. (2015). Fire behaviour of thermally insulated RC beams strengthened with NSM-CFRP strips: Experimental study. Compos. B Eng..

[25] Scarcia W., Palma G., Falconi M.C., de Leonardis F., Passaro V.M.N., Prudenzano F. (2015). Electromagnetic Modelling of Fiber Sensors for Low-Cost and High Sensitivity Temperature Monitoring. Sensors.

[26] Sakairi Y., Uchiyama H., Li Z.M., Adachi S., Rao Y.J., Jones J.D., Naruse H., Chen R.I. (2002). A system for measuring temperature and strain separately by BOTDR and OTDR. Advanced Sensor Systems and Applications.

[27] Kwon I.B., Kim C.Y., Choi M.Y., Liu S.C. (2003). Distributed strain and temperature measurement of a beam using fiber optic BOTDA sensor. Smart Structures and Materials 2003: Smart Systems and Nondestructive Evaluation for Civil Infrastructures.

[28] Ruffle B., Ayrinhac S., Courtens E., Vacher R., Foret M., Wischnewski A., Buchenau U. (2010). Scaling the temperature-dependent boson peak of vitreous silica with the high-frequency bulk modulus derived from Brillouin scattering data. Phys. Rev. Lett..

[29] De Faoite D., Browne D.J., Chang-Díaz F.R., Stanton K.T. (2011). A review of the processing, composition, and temperature-dependent mechanical and thermal properties of dielectric technical ceramics. J. Mater. Sci..

[30] Bucaro J.A., Dardy H.D. (1974). High-temperature Brillouin scattering in fused quartz. J. Appl. Phys..

[31] Hong S.Y., Kim Y.C., Wang M., Kim H.-I., Byun D.-Y., Nam J.-D., Chou T.-W., Ajayan P.M., Ci L., Suhr J. (2018). Experimental investigation of mechanical properties of UV-Curable 3D printing materials. Polymer.

